# Genome-Wide Identification of *LBD* Genes in Foxtail Millet (*Setaria italica*) and Functional Characterization of *SiLBD21*

**DOI:** 10.3390/ijms24087110

**Published:** 2023-04-12

**Authors:** Kunjie Li, Yaning Wei, Yimin Wang, Bin Tan, Shoukun Chen, Haifeng Li

**Affiliations:** College of Agronomy, Northwest A&F University, Yangling, Xianyang 712100, China

**Keywords:** *LBD*, foxtail millet, root development, over-expression, functional characterization

## Abstract

Plant-specific lateral organ boundaries domain (LBD) proteins play important roles in plant growth and development. Foxtail millet (*Setaria italica*) is one new C_4_ model crop. However, the functions of foxtail millet *LBD* genes are unknown. In this study, a genome-wide identification of foxtail millet *LBD* genes and a systematical analysis were conducted. A total of 33 *SiLBD* genes were identified. They are unevenly distributed on nine chromosomes. Among these *SiLBD* genes, six segmental duplication pairs were detected. The thirty-three encoded SiLBD proteins could be classified into two classes and seven clades. Members in the same clade have similar gene structure and motif composition. Forty-seven kinds of *cis*-elements were found in the putative promoters, and they are related to development/growth, hormone, and abiotic stress response, respectively. Meanwhile, the expression pattern was investigated. Most *SiLBD* genes are expressed in different tissues, while several genes are mainly expressed in one or two kinds of tissues. In addition, most *SiLBD* genes respond to different abiotic stresses. Furthermore, the function of *SiLBD21*, which is mainly expressed in roots, was characterized by ectopic expression in *Arabidopsis* and rice. Compared to controls, transgenic plants generated shorter primary roots and more lateral roots, indicating the function of *SiLBD21* in root development. Overall, our study laid the foundation for further functional elucidation of *SiLBD* genes.

## 1. Introduction

Lateral organ boundaries domain (LBD) transcription factors (TFs), also named AS2/LOB [1,2], are characterized by an N-terminal LOB domain, which contains three specific motifs: CX_2_CX_6_CX_3_C, GAS-block, and LX_6_LX_3_LX_6_L. LBD TFs were classified into classes I and II [3,4]. Most class I members contain one zinc finger motif CX_2_CX_6_CX_3_C, one GAS-block motif, and one LX_6_LX_3_LX_6_L spiral coiled structure [5,6]. Motif CX2CX6CX3C is responsible for DNA binding [7]; motif GAS-block is associated with the biological function [8]; leucine-zipper-like coiled-coil motif LX6LX3LX6L is essential for protein dimerization [9]. Compared to class I, class II members only contain one motif similar to the zinc finger CX_2_CX_6_CX_3_C [9]. Many LBD TFs have been identified in different species, including 43 members in *Arabidopsis* [5], 36 in rice (*Oryza sativa*) [3], 90 in *Glycine max* [10], 44 in *Zea mays* [11], 90 in *Triticum aestivum* [12], 28 in *Brachypodium* [13], 43 in *Solanum tuberosum* [14], 126 in *Brassica napus* [15], and so on.

LBD TFs play important roles in plant growth and development [9,16]. In *Arabidopsis*, class I member LBD16 activates the expression of *PUCHI* genes to promote the formation of lateral roots [17]; LBD18 interacts with GIP and activates the expression of *AtEXP14* and *AtEXP17* or forms a heterodimer with AtLBD33 to induce the expression of cell cycle transcription activator gene *E2Fa* and promote lateral root formation [18]. In rice, OsDH1 participates in rice floral development [19]; OsARL1, an auxin-responsive LBD TF, is involved in auxin-mediated cell differentiation and controls the initiation of adventitious root primordial [20]. In wheat, TaMOR interacts with ARF5 to induce *PIN2* expression in the root base and regulate crown root initiation [21]. In addition, transgenic *Arabidopsis* over-expressing *GmLBD12* produced more lateral roots [10]. *MdLBD29*, an apple *LBD* class Ia gene homologous to *AtLBD29*, is activated by MdWOX11 and controls adventitious root formation [22]. Over-expression of the switchgrass (*Panicum virgatum*) class Ic gene *PvLBD12* enhances salt tolerance by altering proline accumulation, malondialdehyde production, K^+^ accumulation, and Na^+^ absorption [23]. *PheLBD29*, a moso bamboo class Ia gene, which is highly expressed in leaves and induced by polyethlene glycol (PEG), may participate in ABA signaling to improve drought tolerance [24]. Compared to class I, the studies on class II are fewer [25]. Over-expression of *OsLBD37* and *OsLBD38* delayed the heading date and increased yield in transgenic rice [26,27]. Over-expression of *MtLBD1*, which encodes a class II protein, also resulted in formation of more lateral roots [28]. *CsLBD39*, a class II gene, is highly expressed in flowers and roots. Ectopic expression of *CsLBD39* in *Arabidopsis* results in smaller rosette leaves, shorter taproots, and fewer lateral roots in plants [29]. The alfalfa (*Medicago sativa*) class II member MsLBD48 inhibits the growth of transgenic *Arabidopsis* by downregulating nitrogen metabolism genes, including *NRT1.1*, *NRT2.1*, *NIA1*, and *NIA2* [30].

Foxtail millet (*Setaria italica*) is one new C_4_ model plant [31] and considered a potential crop for addressing food security issues in the context in the ongoing pandemic crisis [32]. In this study, 33 *SiLBD* genes were identified, and the expression pattern was investigated. Furthermore, the function of *SiLBD21* was characterized by over-expressing in *Arabidopsis* and rice. This study provided some basic information and clues and laid the foundation for further functional elucidation of *SiLBD* genes.

## 2. Results

### 2.1. LBD TFs in Foxtail Millet

**Figure 1:**
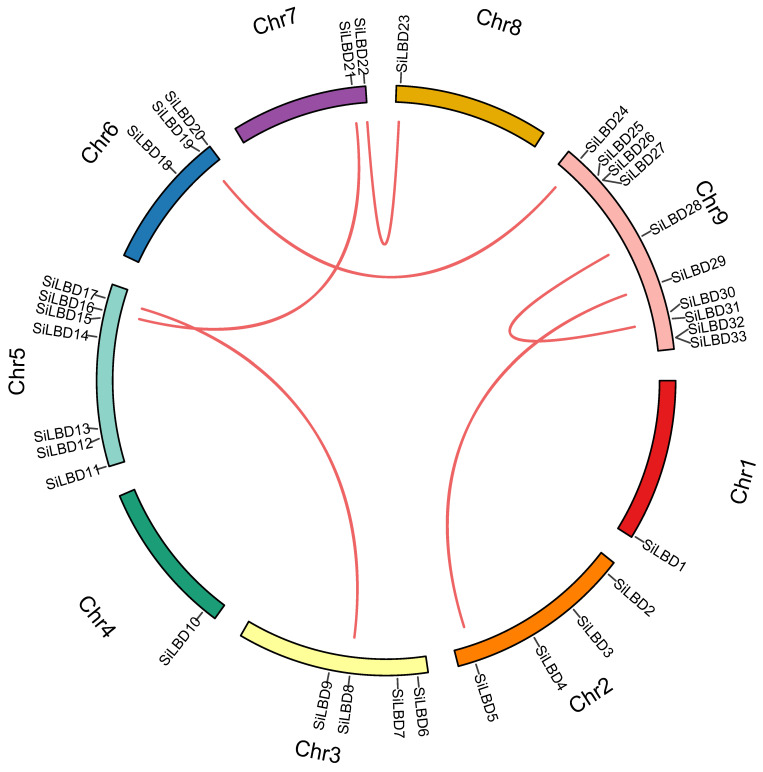
Chromosome location and segmental duplication of *SiLBD* genes. Different chromosomes are shown by different colors, and the gene pairs are indicated by lines.

Thirty-three *SiLBD* genes were identified in the foxtail millet genome (Table S1), representing 0.085% of the annotated foxtail millet genes. They are unevenly distributed on nine chromosomes (Figure 1). For example, there is one *SiLBD* gene on chromosomes 1, 4, and 8, respectively, while there are ten *SiLBD* genes on chromosome 9 (Figure 1). According to the chromosome distribution, 33 genes were numbered as *SiLBD1* to *SiLBD33*. Then, tandem duplication and segmental duplication events were detected. As results, six segmental duplication pairs were identified (Figure 1 and Table S2).

These encoded SiLBD proteins were predicted to localize in the nucleus, suggesting that they function as TFs. The predicted length of these SiLBD proteins ranges from 91 (SiLBD14) to 681 (SiLBD2) amino acids; the predicted molecular weight ranges from 9.96 (SiLBD14) to 72kDa (SiLBD2); and the theoretical isoelectric points range from 5.41 (SiLBD20) to 9.85 (SiLBD24) (Table S1).

### 2.2. Phylogenetic Tree, Gene Structures, and Conserved Motifs

**Figure 2:**
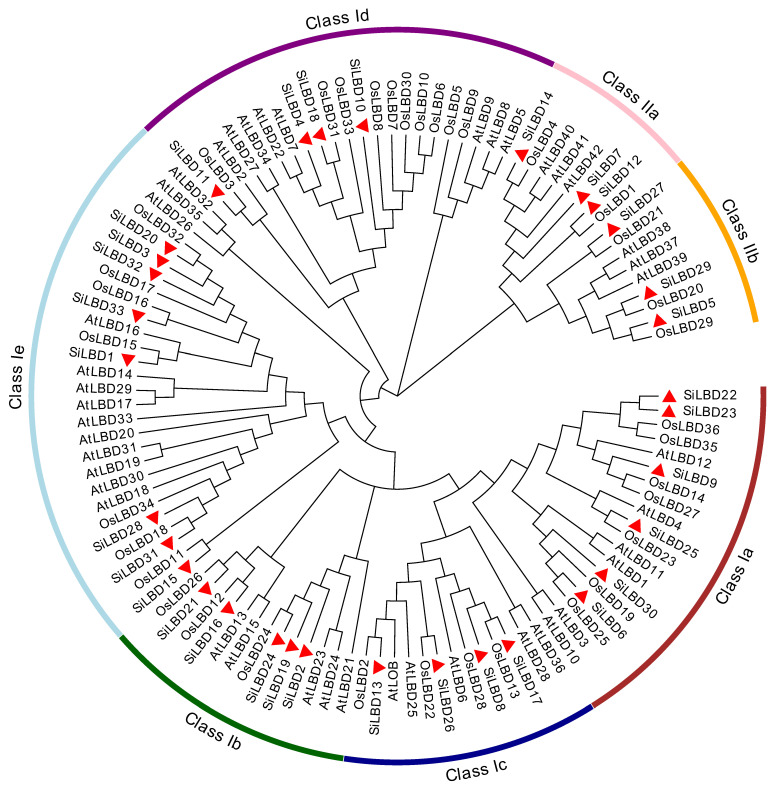
Phylogenetic tree of Arabidopsis thaliana (At), Oryza sativa (Os), and Setaria italica (Si) LBD proteins. SiLBDs are shown by red triangles.

**Figure 3:**
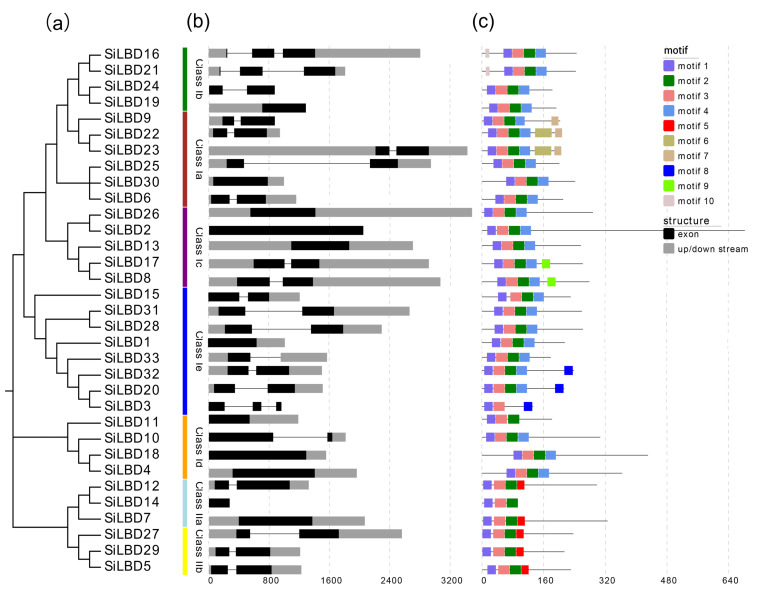
Gene structure and conserved motifs of SiLBDs. (**a**) SiLBDs are classified into seven groups according to bootstrap values; (**b**) gene structures of *SiLBDs*. Exons and introns are indicated by boxes and lines, respectively; (**c**) different motifs of SiLBDs. Different color boxes indicate different motifs.

According to the phylogenetic tree, thirty-three SiLBD TFs were grouped into two classes and seven clades. There are three members in class Ia, eight in class Ib, eight in class Ic, four in class Id, four in class Ie, three in class IIa, and three in class IIb (Figure 2). In addition, the gene structure and conserved protein motifs were analyzed. In general, *SiLBDs* have simple structures: 11 genes just have one exon; 19 genes have two exons; 3 genes have three exons (Figure 3b). A total of 10 motifs were identified in SiLBD proteins (Figure 3c). Motif 1 and motif 3 are present in all SiLBD proteins. Motif 2 is present in 32 SiLBD proteins, except SiLBD3. Motif 1 constitutes CX2CX6CX3C; motif 2 constitutes GAS-block; motif 3 constitutes LX6LX3LX6L (Figure S1). On the contrary, motif 4 is class I specific, and motif 5 is class II specific. 

### 2.3. Cis-Elements

**Figure 4:**
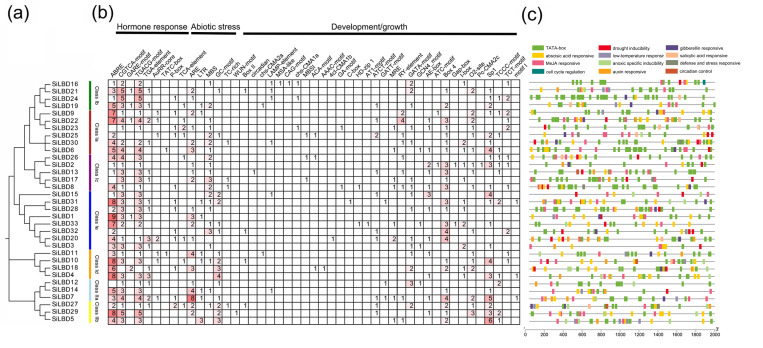
Identified cis-elements in putative *SiLBD* genes’ promoters. (**a**) Phylogenetic tree of 33 *SiLBD* genes. (**b**) Three types of cis-elements identified in the *SiLBD* genes’ promoters. The description on the top represents the predicted cis-elements in the promoter regions and tabulated numbers with different colors indicate the number of cis-elements. (**c**) Locations of 11 classes of cis-elements in *SiLBD* genes’ promoter region.

*Cis*-elements in gene promoter regions are associated with the expression pattern. Therefore, they were identified in putative *SiLBD* gene promoters (2k-bp upstream sequence). A total of 1566 *cis*-elements and 48 kinds were detected (800 specific *cis*-elements and 766 TATA-box elements) (Table S5). They were grouped into three types and twelve classes: hormone response (400), development/growth (280), and abiotic stress response (120) (Figure 4b,c). *Cis*-elements belonging to 11 classes *(cis*-elements related to light responsiveness were too many to be shown) were visualized according to their response characteristics and location (Figure 4c). The TATA-box is one of the components that make up the eukaryotic promoter [33]. It determines the start of gene transcription [34] and is one of the binding sites of the polymerase. Transcription cannot begin until the polymerase is firmly bound to the TATA-box [35,36]. Therefore, we identified the TATA-box element in the promoter region, which is largely present in the promoter region of the *SiLBD* genes (Table S5 and Figure 4c), suggesting that the expression of the *SiLBD* genes is regulated by transcription factors. The hormone response elements include nine kinds of *cis*-elements. The most frequent one is ABRE, which is present in 30 *SiLBD* gene promoters 180 times. Additionally, 19 *SiLBD* genes contain gibberellin (GA)-response element GARE-motif [37] and P-box [38]; 17 *SiLBD* genes contain auxin-response element TGA-element [39] and AuxRR-core [40]. Meanwhile, MeJA and salicylic acid-response elements, such as TGACG-motif [41] and TCA-element [42], were also found, suggesting that *SiLBD* genes participated in the response to hormones extensively. Development/growth *cis*-elements includes 32 kinds of *cis*-elements, such as Sp1 (GGGCGG), a light-response element, root regulation *cis*-element G-box [43], and the metabolism regulation *cis*-element O2-site (GATGATGTGG) [44]. Abiotic stress-response elements include six kinds of *cis*-elements, such as LTR (CCGAAA) [45], which responds to low temperature.

### 2.4. Expression Profiles

**Figure 5:**
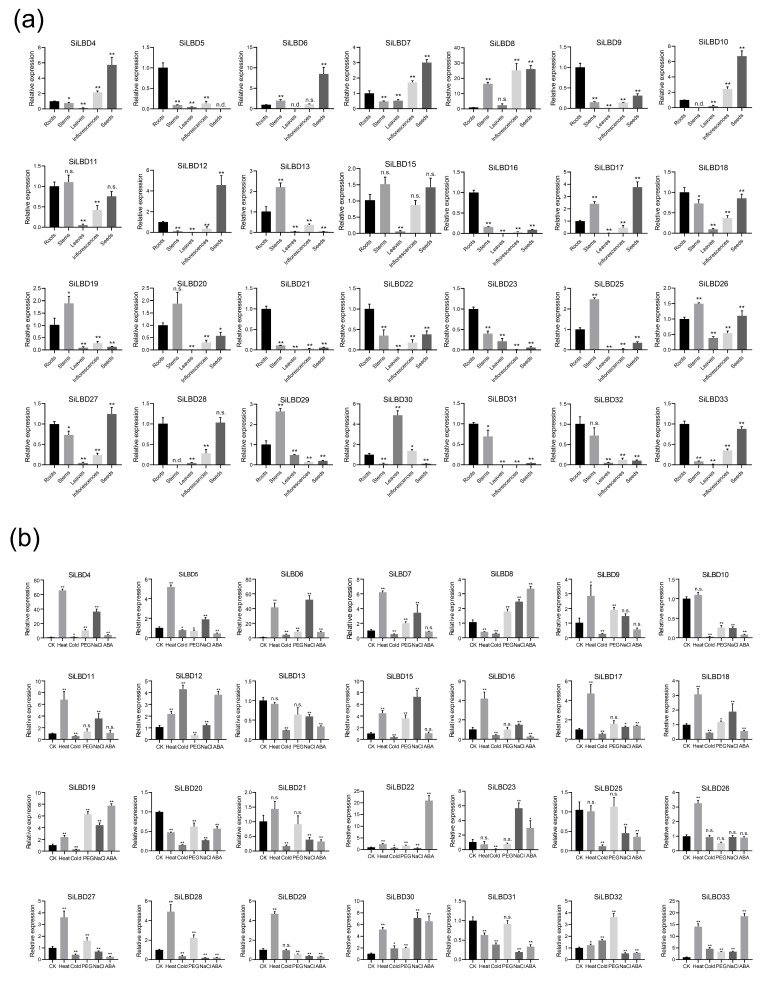
The expression patterns of *SiLBD* genes. (**a**) Expression patterns of *SiLBD* genes in different organs. (**b**) Expression patterns of *SiLBD* genes in 2-week seedlings treated by different stresses. CK in (**b**) indicates control. Error bars represent standard deviations, and statistically significant difference are indicated: ‘*’, 0.01 < *p* ≤ 0.05; ‘**’, *p* ≤ 0.01; ‘n.d.’, no detection; ‘n.s.’, no significance (Student’s *t*-test).

Since the temporal and spatial expression pattern of genes is closely related to the function, we analyzed the expression of *SiLBD* genes by performing qRT-PCR. In general, the expression of twenty-eight genes was detected, while the expression of the other five genes could not be detected when three different pairs of primers were used. Most of the expressed genes are widely expressed in roots, stems, leaves, inflorescences, and seeds, such as *SiLBD7*, *SiLBD11*, *SiLBD15*, *SiLBD17*, *SiLBD18*, and so on (Figure 5a). On the contrary, some genes are mainly expressed in one or two organs. For example, *SiLBD5*, *SiLBD9*, *SiLBD16*, *SiLBD21*, *SiLBD22*, *SiLBD23* are mainly expressed in roots; *SiLBD12* is mainly expressed in seeds (Figure 5a). Noticeably, 13 genes are expressed in roots at a high level, indicating that *SiLBD* genes play important roles in root development.

In addition, the expression in 2-week seedlings treated with different abiotic stresses was also analyzed (Figure 5b). Among 28 expressed genes, the expression of 13 genes was induced by heat; the expression of 9 genes was upregulated by NaCl. Since many ABRE elements were found in the promoters of *SiLBD* genes (Figure 4), we also analyzed the effect of ABA on gene expression. As a result, the expression of five genes, *SiLBD8*, *SiLBD12*, *SiLBD19*, *SiLBD22*, and *SiLBD33*, was upregulated by exogenous ABA (Figure 5b). These results indicated that *SiLBD* genes might play some roles in response to abiotic stresses.

### 2.5. Phenotypes of Transgenic Arabidopsis and Rice Over-Expressing SiLBD21

**Figure 6:**
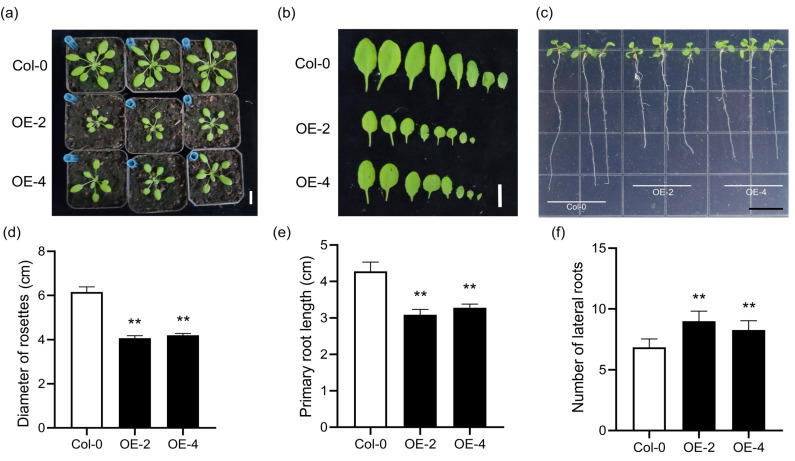
Phenotypes of transgenic *Arabidopsis* over-expressing *SiLBD21*. (**a**) Three-week-old WT and transgenic plants (bar = 2 cm). (**b**) WT and transgenic leaves in (**a**) (bar = 1 cm). (**c**) Root phenotype of 10-day WT and transgenic seedlings (bar = 1 cm). (**d**) Diameter of rosette leaves (n > 15). (**e**,**f**) The length of primary roots and the number of lateral roots (n > 30). Error bars represent standard deviations. Significant differences are indicated: ‘**’, *p* ≤ 0.01 (Student’s *t*-test).

*SiLBD21* is mainly expressed in roots (Figure 5a), and we speculate that it might be involved in root development. To verify this, *SiLBD21* was over-expressed in *Arabidopsis* and rice. Two transgenic *Arabidopsis* and rice lines with high expression were selected for phenotype analyses (Figure S2). On the one hand, transgenic *Arabidopsis*’ rosette leaves were much smaller. The average diameter of 3-week-old transgenic rosette leaves was 3.91 ± 0.07 cm (OE-2) (n = 14), 4.08 ± 0.04 cm (OE-4) (n = 15), significantly narrower than 6.12 ± 0.23 cm (n = 15), that of WT (Figure 6b). On the other hand, the transgenic seedlings had shorter primary roots. The average length of 10-day transgenic primary roots was 3.02 ± 0.15 cm (OE-2) (n = 30), 3.17 ± 0.16 cm (OE-4) (n = 30), significantly shorter than 4.23 ± 0.32 cm (n = 30), that of WT. On the contrary, more lateral roots were generated in transgenic *Arabidopsis* (Figure 6c,e,f). The average lateral root number of transgenic seedlings was 8.04 ± 1.84 (OE-2) (n = 30), and 7.67 ± 1.87 in one primary root, significantly more than that of WT (6.51 ± 0.14) (n = 30).

**Figure 7:**
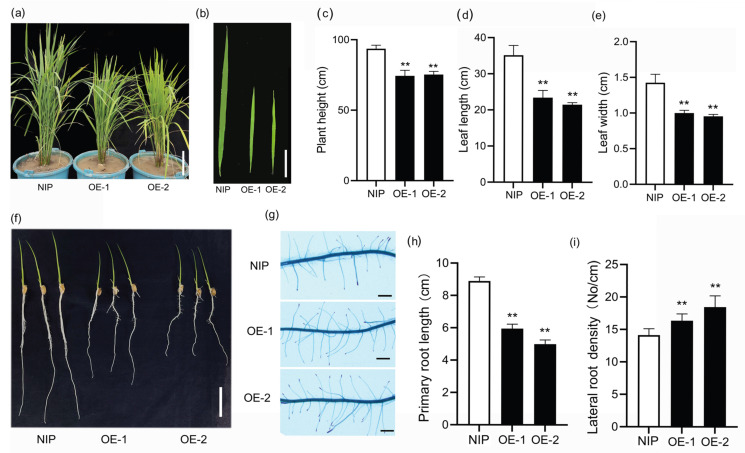
Phenotypes of transgenic rice over-expressing *SiLBD21*. (**a**,**b**) The plant architecture and leaves at the grain-filling stage. (**c**–**e**) Plant height, leaf length and width at the grain-filling stage, n > 10. (**f**) Seven-day WT and transgenic plants’ root length. (**g**) Primary root zones in (**f**) stained with methylene. (**h**,**i**) Primary root length and lateral root density of 7-day WT and transgenic lines, n > 30. Error bars represent the standard deviations. Statistically significant differences are indicated: ‘**’, *p* ≤ 0.01 (Student’s *t*-test). Bars = 10 cm in (**a**,**b**), 2 cm in (**f**,**g**).

Similar phenotypes were observed in transgenic rice (Figure 7). First, the height of transgenic rice was decreased (Figure 7a,c), and the average height of OE-1 and OE-2 plants was 77.01 ± 4.23 cm (n = 12) and 79.21 ± 2.46 cm (n = 13), respectively, significantly shorter than that of WT, 92.41 ± 2.14 cm (n = 15). Second, the transgenic leaves were shorter and narrower than controls (Figure 7b,d,e): the leaf length of OE-1 and OE-2 was 23.12 ± 2.24 cm (n = 15) and 20.46 ± 0.71 cm (n = 15), respectively, while that of the control was 35.74 ± 3.45 cm (n = 15); the leaf width of OE-1 and OE-2 was 1.02 ± 0.42 cm (n = 15) and 0.97 ± 0.27 cm (n = 15), while that of the control was 1.42 ± 0.71 cm (n = 15). Third, at the one-week seedling stage, the primary roots of OE-1 and OE-2 were 5.94 ± 0.27 cm (n = 30) and 4.96 ± 0.28 cm (n = 31) in length, respectively, and were shorter than the control (8.67 ± 0.18 cm) (Figure 7f,h) (n = 33). However, the density of lateral roots was significantly higher than the control (Figure 7g,i).

## 3. Discussion

*LBD* genes are widely involved in plant growth and stress response [25,46]. However, there is no relevant literature to report the function of *SiLBD* genes in foxtail millet, a novel C_4_ model crop. Therefore, there is a need to conduct systematic research on the *SiLBD* genes to fill the gap. Here, 33 SiLBD members were identified, and the gene structure, conserved motifs, cis-elements, and gene expression patterns were analyzed. Meanwhile, a root-specific expression gene, *SiLBD21*, was heterologously transformed into the dicotyledonous model plant *Arabidopsis* and monocotyledonous model crop rice, demonstrating that the function of the *LBD* gene in plant growth and development is conducive to understanding the function of the *LBD* gene in foxtail millet.

Among thirty-three SiLBD TFs, twenty-seven (81.82%) belong to class I and six (18.18%) belong to class II. Obviously, there are many more member in class I than in class II, which is consistent with previous studies [47,48,49]. The phylogenetic tree analysis of LBD genes in species including *Arabidopsis* and rice showed that *LBD* genes were conserved during plant evolution. In addition, SiLBD27 is close to AtLBD37, AtLBD38, and AtLBD39 in class IIb, which means that this foxtail millet *LBD* gene may have similar biological functions to the three class IIb members of *Arabidopsis*, which may affect nitrogen response and metabolic pathways [50]. Therefore, editing of this *LBD* gene may improve the efficiency of genetic modification.

SiLBDs in adjacent branches have similar gene structure and conserved motifs, which suggests that they may have similar biological functions. Almost all genes have cis-elements that respond to MeJA and abscisic acid, indicating that the promoters of the *SiLBD* gene are conserved and that SiLBDs may be involved in abiotic stress. Few *SiLBD* genes contain cis-elements of GA- and SA-response elements, suggesting that these SiLBDs may participate in plant cell division and pathogenic immunity.

*SiLBD* genes had the characteristics of tissue expression specificity and stress response [9]. We performed a qRT-PCR assay on 28 *SiLBD* genes to analyze the expression pattern. Among the genes specifically expressed in five tissues (roots, stems, leaves, inflorescences, and seeds), the root-specific genes are the most abundant (6/28) (Figure 5a), suggesting that these genes may be closely related to root development. The expression of *LBD* genes is simultaneously up- or downregulated by simulated abiotic stress treatments (Figure 5b), suggesting that they may respond to abiotic stress. At the same time, some genes in the same phylogenetic branch have similar expression patterns, such as *SiLBD9*, *SiLBD22*, and *SiLBD23*, genes of class Ia, which are highly expressed in the root; class IIb genes *SiLBD5*, *SiLBD27*, and *SiLBD29* were upregulated after heat treatment, suggesting that they may have similar biological functions in response to heat stress.

### 3.1. Monocot Plants Had Fewer LBD Genes

The rice genome (diploid, 466 Mb) [51] is much larger than the *Arabidopsis* genome (diploid, 123 Mb) [52], but there are more *Arabidopsis LBD* genes (43 *AtLBDs*) than rice *LBD* genes (36 *OsLBDs*). Similar to *Arabidopsis*, most dicot plants have more *LBD* genes. For example, soybean (*Glycine max*, diploid, 1.0 Gb) has 90 *GmLBDs* [53], oil-seed rape (*Brassica napus*, tetraploid, 1.1 Gb) has 126 *BnLBDs* [54], and cotton (*Gossypium hirsutum,* tetraploid, 2.4 Gb) has 131 *GhLBDs* [55,56]. On the contrary, monocot plants have fewer *LBD* genes. For example, maize (diploid, 2.3 Gb) has 44 *ZmLBDs* [57]; wheat (hexaploid, 14.5 Gb) *has* 90 *TaLBDs* [58]; *Brachypodium* (diploid, 260 Mb) has 28 *BdLBDs* [59]. In this study, we found that foxtail millet (diploid, 515 Mb) has 33 *SiLBDs.* Since gene duplication is the main driver of gene expansion and evolution [60], we identified duplication events in the plants mentioned above. We found 36, 77, 93, and 60 gene duplication events in the dicot *Arabidopsis*, *Glycine max*, *Brassica napus*, and cotton, respectively (Table S3) [10,15,55]. Meanwhile, in monocots, there are only 6, 4, 11, 38, and 10 gene duplication events in foxtail millet, rice, maize, wheat, and *Brachypodium*, respectively (Table S4) [11,12,13]. Most of the *LBD* gene duplication events mentioned above are segmental duplication. These results indicated that the number difference between dicot and monocot *LBD* genes might result from gene duplication.

### 3.2. LBD TFs Display Extensive Functions

LBD TFs are plant specific and involved in various biological processes, such as lateral organ development, the establishment of plant polar growth, and the nitrogen metabolism pathway [25]. The functions of many LBD TFs have been reported in different species, such as AtLOB/ASL4 [61], AtLBD3/ASL9 [62], OsLBD37, and OsLBD38 [27]. These studies showed extensive functions of LBD TFs. For example, three class IIb members, *AtLBD37*, *AtLBD38*, and *AtLBD39*, act as negative regulators of anthocyanin biosynthesis and participate in nitrogen metabolism [50,63]. Class Ia members *AtLBD6/AS2*, *AtLBD36/AS1*, and *TaAS2* regulate the polarity of leaf adaxial–abaxial growth [64,65,66]. Recently, *ZmLBD5* was reported to negatively regulate drought tolerance [67], and over-expression of *CsLOB1*, a citrus *LBD* gene, was shown to cause increased susceptibility to citrus bacterial canker (CBC) disease [68]. This expands our understanding of the biological functions of LBD TFs in response to stresses.

On the one hand, most *SiLBD* genes are expressed in different organs and respond to different abiotic stresses (Figure 5). On the other hand, 47 kinds of *cis*-elements were identified in *SiLBD* putative promoters. They are involved in development/growth, hormone signaling, and abiotic stresses (Figure 4). These results implied that SiLBD TFs also play various roles.

### 3.3. LBD TFs Play Important Roles in Root Development

Much evidence has shown that LBD TFs play crucial roles in root development. *AtLBD16* and *AtLBD18* are involved in the auxin signal transduction pathway and lead to the formation of lateral roots [69]. *AtLBD14*, downregulated by ABA, is involved in the ABA-mediated control of lateral root formation [70]. *Crl1*, a rice *LBD* gene, which is a target of ARFs in auxin signaling, is essential for crown root formation [71]. *RTCS* encodes a maize LOB domain protein and initiates the embryonic seminal and post-embryonic shoot-borne root system [72]. The wheat LBD TF TaMOR interacts with TaMRRP, leading to more lateral roots in over-expression *Arabidopsis* and more crown roots in over-expression rice [73]. CmLBD1 positively regulates the response to auxin fluctuation and lateral root formation [74]. These results suggest that the *LBD* genes function in root development through different molecular pathways.

Our result is similar to that of over-expression lines of the class Ib member AtLBD13 [75]. Over-expression of *SiLBD21* in *Arabidopsis* and rice resulted in shorter primary roots and more lateral roots, implying the dual functions in root development.

## 4. Materials and Methods

### 4.1. Genome-Wide Identification of SiLBD Genes

The LBD TFs in *Arabidopsis* and rice were described in previous research [3,6]. First, homologous protein alignment was performed using BLAST with E-value < e × 10^−10^ and identity >50%, to identify SiLBD proteins in the foxtail millet protein database. Then, a hidden Markov model (HMM) of LBD proteins, LOB domain (PF03159), was downloaded from the Pfam database [76] and used to search against the protein database using HMMER3 software (http://hmmer.org/, accessed on 4 March 2021) with E-value < e × 10^−5^. After integrating the results of the above two steps, a manual correction was performed to remove alternative splicing and redundancy.

### 4.2. Physicochemical Properties, Gene Duplication, Chromosome Distribution, and Phylogenetic Analyses

The physicochemical properties of the proteins were predicted using the website ExPASY [77], and the Plant-mploc web server [78] was used to predict the subcellular localization. The chromosome distribution, coding sequence, genomic sequence, and 2k-bp upstream genomic sequences were obtained from the Phytozome database [79]. Gene duplication events were investigated with Gu’s method [80] and visualized using TBtools software [81]. The phylogenetic tree was constructed using the neighbor-joining(N-J) method [82] with 1000 bootstrap replications.

### 4.3. Gene Structure, Conserved Motif, and Cis-Element Analyses

The exons and introns were displayed graphically on the Gene Structure Display Server (GSDS) [83]. The sequence of SiLBD proteins was submitted to MEME Suite [84] to detect conserved motifs with the following parameters: the maximum number of motifs was 10, and the optimum motif width fell between 6 and 200. The *cis*-elements were predicted on the Plant CARE website [85] by submitting the 2k-bp upstream genomic sequences. According to the function annotation, they were divided into 3 main types and 12 classes and visualized using Excel 2016 (Microsoft, New York, NY, USA) and TBtools. The phylogenetic tree, gene structure, and conserved motifs were visualized by the Evolview website [86].

### 4.4. Plant Growth Conditions and Treatments

Foxtail millet variety ‘*Yugu 18*′ was used in this study and planted under natural conditions (Yangling, China). For expression analysis, the roots, stems, leaves, and inflorescences were collected at the heading stage. For different abiotic stresses analysis, 2-week seedlings were treated with 20% PEG6000, 200 mM NaCl, and 100µM abscisic acid (ABA) at 42 °C and 4 °C for 2 h, respectively. *Arabidopsis* was planted in an artificial greenhouse with a 16 h/8 h (day/night) photoperiod at 22 °C/18 °C; rice was planted in an experimental plot under natural conditions (Yangling, China).

### 4.5. RNA Extraction and qRT-PCR Analysis

Total RNAs were extracted using a TRIZOL reagent (TAKARA) according to the protocol. The cDNAs were synthesized using an Evo M-MLV RT Kit (Accurate Biology). Fifteen microliters of the qRT-PCR reaction system was configured using the SYBR^®^ Green Premix Pro Taq HS qPCR Kit (Accurate Biology, Changsha, China), which contained 7.5 µL SYBR mix, 0.5 µL cDNA (200 ng µL^−1^), 0.6 µL forward and reverse primers (10 pmol µL^−1^), and 5.8 µL ddH_2_O. The following conditions were set in a QuantStudio5 (Thermo Fisher): 95 °C for 5 min at the pre-denaturation stage, then 95 °C for 15 s and 60 °C for 30 s for 40 cycles in the PCR stage, and 95 °C for 15 s, 60 °C for 1 min, 95 °C for 15 s in the melt-curve stage. The relative expression level calculation was performed using the 2^(−ΔΔCt)^ method [87]. Primers used in this study are listed in Table S6.

### 4.6. Generation of Transgenic Plants

The full-length coding sequence of *SiLBD21* was amplified using foxtail millet root cDNA as a template and cloned into the pCAMBIA1301 vector. The floral dip method was used for *Arabidopsis* (Col-0) transformation [88]. Rice (Nipponbare) was transformed according to a previous report [89]. Positive transgenic plants were identified by PCR and the expression level was determined by qRT-PCR, and 2 lines with high expression were selected for further study. Primers used for PCR and RT-PCR are listed in Table S7.

### 4.7. Phenotype Analysis of Transgenic Plants

The root length of 10-day *Arabidopsis* seedlings was measured with a ruler, and the number of lateral roots in the primary root was counted. The diameter of 3-week-old rosette leaves was measured. The root length of 1-week rice seedlings was measured. The roots were stained with methylene blue and the number of lateral roots was counted with an LA-S Plant Root Analyzer System. Plant height and leaf length and width of rice were measured at the grain-filling stage.

### 4.8. Data Processing and Analysis

All data were statistically analyzed and visualized using GraphPad Prism 8.0 software (San Diego, CA, USA), with three replicates set for each group and significant differences between data expressed as ‘*’ (0.01 < *p* ≤ 0.05) or ‘**’ (*p* ≤ 0.01) according to the Student’s *t*-test.

## 5. Conclusions

With the update of foxtail millet genome sequencing and the establishment of a transformation system, foxtail millet is gradually becoming a new C_4_ model crop, however, no study has reported about the foxtail millet *LBD* genes, which is a gap in the functional research of foxtail millet. To complement this research, we identified and classified the members of the SiLBD family for the first time. Here, 33 *SiLBD* genes were identified. These encoded SiLBD TFs were divided into two classes and seven clades. Three types and forty-eight kinds of *cis*-elements were identified in the putative promoters. The qRT-PCR experiments were performed on different organs and different abiotic stresses to study the expression pattern of *SiLBD* genes. Most *SiLBD* genes are expressed extensively and induced by abiotic stresses. These results indicated that SiLBD TFs perform several functions. Over-expressed *SiLBD21* in transgenic *Arabidopsis* and rice showed functions in root development.

## Data Availability

Not applicable.

## Supplementary Materials

The supporting information can be downloaded at: https://www.mdpi.com/article/10.3390/ijms24087110/s1.
