# Supplementary material for: Genome-Wide Identification of LBD Genes in Foxtail Millet (Setaria italica) and Functional Characterization of SiLBD21
Source: Int J Mol Sci. 2023 Apr 12;24(8):7110. doi: 10.3390/ijms24087110 (PMC10138450; doi:10.3390/ijms24087110)
Supplement: Supplementary file 1 [file ijms-24-07110-s001.zip › Figure S1.pdf]

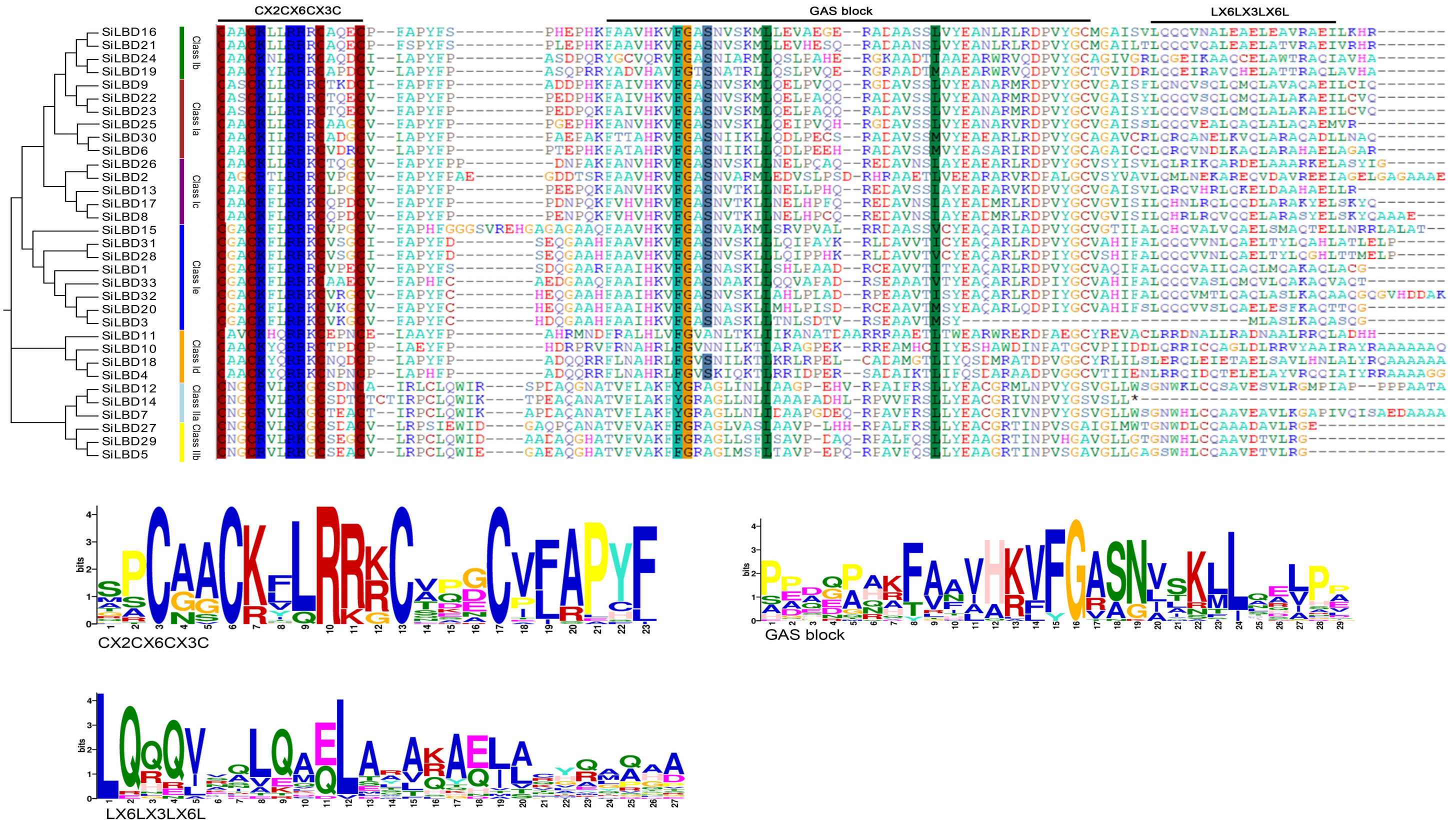

Figure S1. Comparison of protein sequence of SiLBDs conserved domains and CX2CX6CX3C motif, GAS motif LX6LX3LX6L motif of SiLBDs.
