# Supplementary material for: Genome-Wide Identification of LBD Genes in Foxtail Millet (Setaria italica) and Functional Characterization of SiLBD21
Source: Int J Mol Sci. 2023 Apr 12;24(8):7110. doi: 10.3390/ijms24087110 (PMC10138450; doi:10.3390/ijms24087110)
Supplement: Supplementary file 1 [file ijms-24-07110-s001.zip › Figure S2.pdf]

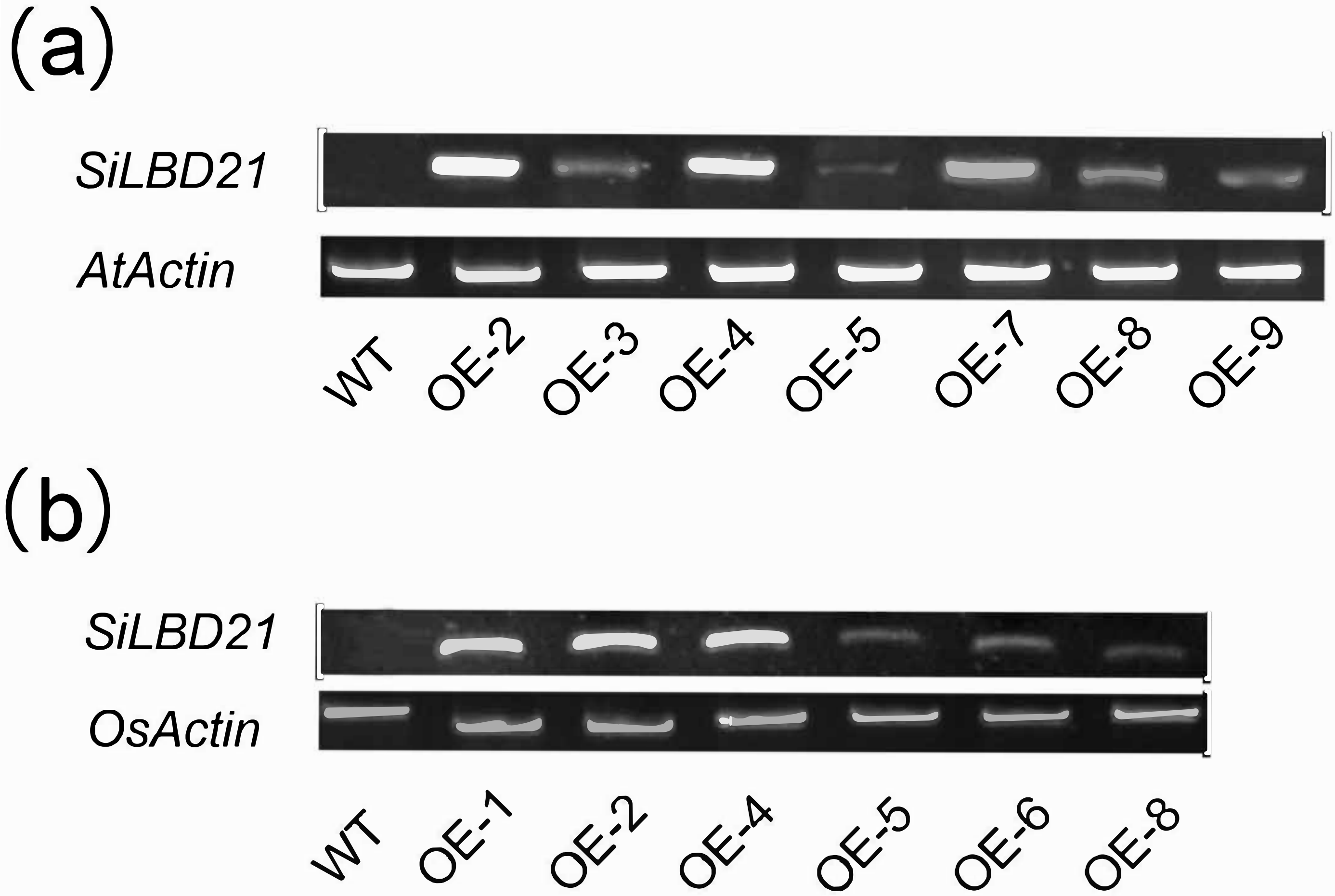

Figure S2. RT-PCR of postive *SiLBD21* transgenic *Arabidopsis* and rice lines. (**a**) 32 cycles of *AtActin*, 30 cycles of *SiLBD21*. (**b**) 30 cycles of *OsActin* and *SiLBD21*.
